# Improving career readiness in middle school students: a systematic review of intervention approaches

**DOI:** 10.3389/fpsyg.2025.1582195

**Published:** 2025-05-08

**Authors:** Danqi Wang, Gang Wang

**Affiliations:** School of Education, Huainan Normal University, Huainan, China

**Keywords:** career intervention, middle school students, theoretical framework, evaluation, systematic review

## Abstract

**Introduction:**

To address the challenges posed by the rapidly evolving career landscape on adolescent development, this study systematically reviews career intervention pathways for middle school students.

**Methods:**

It explores the theoretical frameworks, intervention structures, evaluation systems, and outcomes associated with career interventions at middle school educational stage. Seven key terms, three databases, and four eligibility criteria were established for the review. Out of 417 articles collected, 21 were selected for comprehensive analysis.

**Results:**

The results indicate that the theoretical framework for middle school career interventions includes career theory and psychotherapy, with the main intervention approach group-based. The evaluation system typically relies on quasi-experimental pre- and post-test assessments. The outcomes demonstrate positive developments in career decisionmaking and career adaptability.

**Discussion:**

This study emphasizes the critical role of career interventions and educational programs during middle school, advocating for their integration into the curriculum to support students’ career development.

## Introduction

Since the 21st century, the acceleration of globalization, the shortening of technological iteration cycles, the normalization of economic fluctuations, and the trend toward labor market flexibility, combined with the volatility, uncertainty, complexity, and ambiguity of the times, have collectively shaped a highly uncertain landscape for career development (Savickas et al., 2009; Sowa et al., 2023). Career trajectories are increasingly unpredictable, and the boundaries of the professional world are becoming more blurred. This presents a significant challenge to the career decision-making abilities and career adaptability of young people. Against this backdrop, the middle school stage (ages 12–15) serves as a critical transitional period in career development, where the effectiveness of educational interventions directly impacts the quality of an individual’s lifelong career development.

According to Super (1990) career development stage theory, the middle school stage represents a key turning point in the transition from the growth stage to the exploration stage. The developmental tasks during this stage focus on the orientation of interest areas and the initial construction of career competencies. Through systematic educational interventions, it is possible to effectively promote the formation of career self-efficacy (Radcliffe and Bos, 2013). Empirical research shows that the career cognitive framework formed during middle school not only influences academic persistence during high school (Pambudi et al., 2019), but is also significantly positively correlated with career satisfaction in adulthood (Raciti and Dale, 2019). The Association for Career and Technical Education (2018) has further identified middle school as a core period for career awareness initiation, career knowledge accumulation, and the preliminary establishment of career goals.

However, there is a significant gap in both educational practices and research: On one hand, studies show that most middle school students have unclear career perceptions, misjudge skill demands, and experience a disconnect between education and career (Babarović et al., 2020). On the other hand, existing career intervention programs mainly focus on high school and higher education stages, with insufficient systematic intervention coverage at the middle school level (Langher et al., 2018; Wang et al., 2024). This structural contradiction leads to two serious consequences: first, students face career selection pressure too early without adequate decision-making support systems (Kudysheva et al., 2024). Second, regional disparities in the allocation of educational resources result in fragmented career education, with insufficient long-term effectiveness and a lack of sufficient attention from educational administrators to middle school career counseling (Van Hai et al., 2022).

In response, this study employs a systematic review method to analyze career intervention research at the middle school level published in internationally recognized databases over the past decade (2015–2024). By establishing a multidimensional evaluation framework, the study aims to reveal the effectiveness boundaries of current intervention models and provide evidence-based support for the construction of a middle school career education paradigm suited to the characteristics of the VUCA era. Most importantly, this research seeks to systematically evaluate the effectiveness of current middle school career education interventions to address the structural changes in the professional world under the backdrop of globalization.

## Methods

The eligibility criteria for selecting studies followed the Population, Intervention, Comparison, Outcome, and Study Design (PICOS) framework (Vashisht et al., 2021). Studies were included based on the following criteria: (a) the population consisted of middle school students; (b) the intervention involved career courses or career counseling; (c) studies in which no comparator groups were required; (d) the focus was on the impact of the intervention on career-related outcomes; and (e) the study employed a quantitative research design, either cross-sectional or longitudinal.

The titles and abstracts of all articles were analyzed independently by two researchers to minimize bias. The objective was to determine whether the study involved middle school students as participants and whether it addressed career intervention and assessment systems. This step constituted the first filtering process. Subsequently, the same researchers independently read the articles that passed the initial screening, applying the same criteria. Ultimately, only the articles that met these standards were retained. Discrepancies between the researchers were resolved through discussion, and any remaining disagreements were settled by a third researcher.

This study conducted a systematic literature review following the PRISMA guidelines (Moher et al., 2015) to explore the theoretical framework, intervention structure, evaluation methods, and outcomes of career interventions for middle school students.

### Databases

The study involved three databases: Web of Science (Clarivate), ProQuest, and ERIC. The inclusion criteria were academic articles published between 2015 and 2024, with English abstracts. The year 2015 was chosen as the starting point to capture the most recent developments in middle school career intervention in the 21st century.

### Eligibility criteria

The inclusion criteria were based on four factors: language, publication year, sample, and description of career interventions. Specifically, only academic articles published in English between 2015 and 2024 that described career interventions for middle school students were considered. If an article was inaccessible, efforts were made to contact colleagues at other universities to gain broader database access.

Following Soares et al. (2022) recommendation, a two-step search process was employed. Relevant search terms were derived from the Elsevier database and discussions with subject and methodology experts. Two researchers independently screened article titles and abstracts to determine whether the study focused on middle school samples and included career intervention and evaluation systems (Soares et al., 2022). Search filters included a date range (2015–2024), article type (academic articles), and Boolean operators (AND, OR) to combine search terms: “career program” OR “career education” OR “career curriculum” OR “career intervention” OR “career guidance” AND “middle school students” OR “junior high school students.”

### Exclusion criteria

Exclusion criteria were adapted from Damodar et al. (2024) and included the following: (a) studies in non-article formats, such as dissertations, conference proceedings, or book chapters; (b) non-English language studies; (c) studies focusing on populations other than middle school students, or participants outside the 12–15 age range; (d) studies lacking group-based intervention designs or validation. After applying these exclusion criteria, relevant articles were summarized according to themes, including theoretical frameworks, intervention structures, evaluation systems, and intervention outcomes (Damodar et al., 2024).

## Results

**Figure 1:**
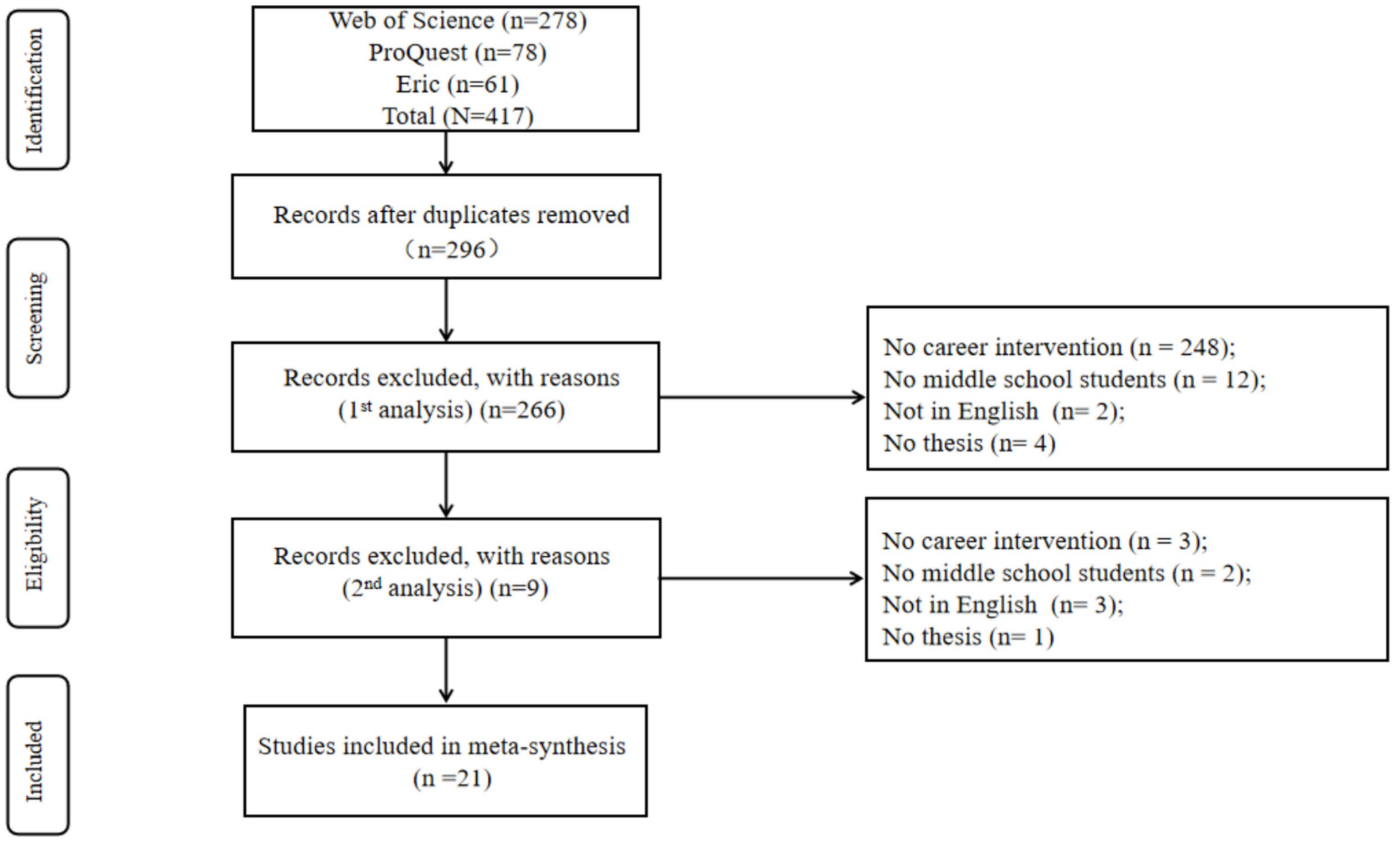
PRISMA flow diagram.

A total of 417 articles were identified through the database search. These articles were first exported to Microsoft Excel to remove duplicates, leaving 296 articles for further screening. The titles and abstracts of these articles were then reviewed. The first round of screening assessed whether the articles met the eligibility criteria. In cases of uncertainty, the articles were moved to the second round of screening. Out of the 296 non-duplicate articles, 248 articles that did not include career interventions, 12 articles without middle school samples, 2 non-English articles, and 4 non-academic papers were excluded in the first round. A total of 30 articles advanced to the second round, where full-text reviews were conducted. Nine articles were excluded in this phase (see Figure 1), and ultimately, 21 articles were included in the systematic review (see Table 1).

**Table 1:** Intervention characteristics.

Study	Intervention structure	Theoretical framework	Evaluation	Outcomes
Glessner et al. (2017)	Online career workshop and college visit (4 days)	SCCT and self-efficacy theory; online Florida CHOICES program	Quasi-experimental; pre- post-test	College and career self-efficacy
Ali et al. (2017)	Workshop (6 sessions)	SCCT; project HOPE	Quasi-experimental; pre- post-test	Math/science self-efficacy, health science career interests
Babarović et al. (2020)	Workshop (8 sessions)	Career development school program and parent’s guide	Quasi-experimental; pre- post-test	Career information and career decision-making
Ali et al. (2019)	Workshop (8 sessions)	SCCT and sociopolitical development; project HOPE	Quasi-experimental; pre- post-test	Health-care career and math/science interests
Nota et al. (2016)	Online career intervention,(3 sessions)	Career construction theory and 1, 2, 3…. future! program	True-experimental; pre- post-test	Career adaptability, life satisfaction and narratives future aspirations
Santilli et al. (2019)	Group career counseling (3 sessions)	Career construction theory and my career story	Quasi-experimental; pre- post-test	Career adaptability and orientation toward the future
Grigal et al. (2019)	Online career curriculum	Future Quest Island project	Qualitative	Career exploration
Pambudi et al. (2019)	Group career counseling	Modeling technique	True-experimental; pre- post-test	Career adaptability and career decision self-efficacy
Edirmanasinghe and Blaginin (2019)	School counseling intervention	Self-efficacy theory and youth participatory action research	Qualitative	Future career options skills
Grant et al. (2021)	Small-group counseling (8 sessions)	Relational cultural theory	Qualitative	Self-efficacy in career exploration
Yuen et al. (2022)	Workshop (7 sessions)	SCCT, positive youth development and “SUN Life” Navigation Project	Longitudinal mixed-method quasi-experimental; pre- post-test and qualitative interview	Personal goal-setting, career goal-setting, and the presence of meaning in life
Albritton et al. (2020)	Individual career counseling	Relational cultural theory	Qualitative	Exposure, support, attainment, family, and effort/persistence
Liu et al. (2021)	Psychological courses (6 sessions)	Acceptance commitment therapy	Quasi-experimental; pre-post and 2 months test	Career adaptability and psychological flexibility
Yüzen and Perkmen (2022)	Group career counseling	Multimedia Learning Theory and Cognitive Load Theory	Quasi-experimental; pre- post-test	Career decision-making self-efficacy
Kim et al. (2022)	Career exploration program (10 sessions)	Reality therapy	Quasi-experimental; pre- post-test	Self-esteem and career decision-making self-efficacy
Syeda and Zahid (2024)	Career curriculum (12 sessions)	SCCT and Kolb’s Experiential Learning Cycle	Quasi-experimental; qualitative focus group discussions	STEM career interest, self-efficacy, and knowledge
Rose and Bowen (2021)	Career curriculum (10 sessions)	CareerStart	Quasi-experimental; pre- post-test	High school dropout
Kotkas et al. (2019)	Career curriculum (5 sessions)	STEM careers introducing	Quasi-experimental; pre- post-test	Career aspirations and perceptions of competencies
Isah and Abdullah (2023)	Career intervention program	No specific model reported	Quasi-experimental; pre- post-test	Vocational preference
Malik et al. (2024)	Career guidance course	Career counselor/teacher	Quasi-experimental; pre- post-test	Career decision-making
Muryono (2022)	Microsite-based career guidance programs	Life and career skills	Quasi-experimental; pre- post-test	Career exploration

### Theoretical framework

Among the 21 included articles, one did not provide a detailed theoretical framework supporting the career intervention applied (Isah and Abdullah, 2023). The theoretical frameworks, however, did not reach a consensus. Most articles focused on Self-efficacy Theory (Edirmanasinghe and Blaginin, 2019; Glessner et al., 2017), Social Cognitive Career Theory (Glessner et al., 2017; Ali et al., 2017; Ali et al., 2019; Yuen et al., 2022; Syeda and Zahid, 2024), Career Construction Theory (Nota et al., 2016; Santilli et al., 2019), and relational cultural theory (Grant et al., 2021; Albritton et al., 2020). Additionally, therapeutic approaches such as reality therapy (Kim et al., 2022), psycho-educational therapy (Pambudi et al., 2019), and acceptance commitment therapy (Liu et al., 2021) were also applied.

### Intervention structure

Various intervention designs were discussed in the included articles. The majority referenced the use of group career counseling (Santilli et al., 2019; Grant et al., 2021; Yüzen and Perkmen, 2022), followed by workshops (Ali et al., 2017; Babarović et al., 2020; Ali et al., 2019; Yuen et al., 2022), and career education curricula (Syeda and Zahid, 2024; Rose and Bowen, 2021; Kotkas et al., 2019; Malik et al., 2024). In addition, computer-based interventions (Glessner et al., 2017; Nota et al., 2016; Muryono, 2022) and modular interventions (Grigal et al., 2019; Pambudi et al., 2019) were also used. Career interventions for middle school students positively influenced various career-related outcomes. Online life design interventions were found to enhance career adaptability and life satisfaction (Nota et al., 2016). For rural students, group counseling promoted career exploration (Grant et al., 2021; Ali et al., 2017). Interventions that linked academic content with careers, such as CareerStart, were shown to reduce dropout rates (Rose and Bowen, 2021). Programs targeting math self-efficacy and health science career exploration demonstrated promise in improving students’ self-efficacy and career interests (Ali et al., 2017). Overall, career interventions in middle school education positively influenced students’ career choices, self-efficacy, and future development, underscoring the importance of early career guidance in schools.

The interventions discussed in the included articles covered various programs, including the online Florida CHOICES program (Glessner et al., 2017), Project HOPE (Ali et al., 2017; Ali et al., 2019), Career Development School Program and Parent’s Guide (Babarović et al., 2020), Positive Youth Development and the “SUN Life” Navigation Project (Yuen et al., 2022), Life and Career Skills (Muryono, 2022), and Life Design (Nota et al., 2016; Santilli et al., 2019).

### Evaluation and intervention outcomes

Overall, the studies included qualitative, quantitative, and mixed-method research. The majority of studies were quantitative interventions, including randomized controlled trials and quasi-experimental designs, which employed pre- post-test evaluation systems (Glessner et al., 2017; Ali et al., 2019; Yüzen and Perkmen, 2022; Kim et al., 2022; Kotkas et al., 2019). Qualitative studies primarily used interview methods, such as focus group interviews (Albritton et al., 2020; Grant et al., 2021; Grigal et al., 2019; Edirmanasinghe and Blaginin, 2019). In mixed-method studies, quantitative and qualitative research were conducted separately (Yuen et al., 2022; Syeda and Zahid, 2024). Regarding the follow-up monitoring of intervention outcomes, only one study (Liu et al., 2021) reported conducting such an assessment. However, the results indicated that, 2 months after the intervention, the outcomes had reverted to levels similar to those observed before the intervention. In qualitative studies, four studies (Albritton et al., 2020; Edirmanasinghe and Blaginin, 2019; Grant et al., 2021; Grigal et al., 2019) focused on evaluating students’ perceptions of career exploration and progress in career skill development during the intervention.

The evaluation dimensions and applied tools, along with the results from each study, are outlined in detail below. The most commonly measured outcomes in middle school career interventions were career decision-making, including career decision-making self-efficacy and career decision-making difficulties. Additionally, career adaptability was frequently assessed. Some studies suggest that career decision-making self-efficacy should not be used as the sole measure of intervention effectiveness, especially when evaluations do not include actual decision-making and career transitions (Babarović et al., 2020). Career adaptability has been used to assess the effectiveness of career interventions, possibly due to the lack of updates in existing measurement tools and criticisms regarding career maturity measures for different populations (Whiston et al., 2017). Career interventions in middle school are also related to STEM fields, with outcomes including math/science self-efficacy and health science career interests (Ali et al., 2017; Ali et al., 2019; Syeda and Zahid, 2024).

## Discussion

This systematic literature review aims to summarize the evidence on career interventions for middle school students. A total of 21 articles met the inclusion criteria. These articles were further examined in detail, focusing on theoretical frameworks, intervention structures, evaluation systems, and outcomes.

### Comparing SCCT and CCT in the middle school perspective

According to our findings, most studies adopt either Career Construction Theory (CCT) or Social Cognitive Career Theory (SCCT) as their theoretical framework. Each theory has its strengths and limitations within the middle school context. Middle school students are in a developmental stage where they form self-efficacy beliefs regarding their abilities. Glessner et al. (2017) indicated that interventions targeting career self-efficacy can enhance students’ confidence in their ability to pursue higher education and career pathways. SCCT-based interventions provide a structured approach to assessing career readiness through self-efficacy measurements, making them well-suited for educational settings. However, SCCT primarily emphasizes self-efficacy and outcome expectations while overlooking deeper issues related to career identity and meaning-making, which are crucial in early adolescence. Moreover, middle school students may have limited career exposure, making it difficult for them to develop strong outcome expectations, thereby constraining the effectiveness of SCCT.

The CCT, developed by Savickas (2005), is grounded in the principles of life design and emphasizes that career development is a process of creating personal meaning. Nota et al. (2016) demonstrated that life design interventions help early adolescents develop adaptive and proactive career planning skills, focusing not on fixed career choices but on fostering skills. CCT helps students connect career exploration with their personal values, interests, and life experiences, which is crucial during the stage when identity is still being formed. However, middle school students may find it difficult to engage in the reflective and abstract thinking required to build a career narrative. CCT interventions may require long-term observation to effectively assess their impact. The individualized narrative approach may demand significant time and guidance, which can be challenging to implement in middle school environments. Therefore, future career interventions could consider developing new approaches or improving existing frameworks within this context (see Table 2).

**Table 2:** Comparing SCCT and CCT in the middle school perspective.

	SCCT	CCT
Theoretical focus	Self-efficacy, outcome expectations, goal-setting	Identity construction, meaning-making, career adaptability
Key strengths	Builds confidence and practical skills, measurable outcomes	Encourages personal exploration, adaptability, and self-reflection
Key limitations	Limited attention to career identity	Harder to measure short-term success
Students	Students who need confidence and exposure to career planning	Students who benefit from exploratory, narrative-based approaches

### The key role of counselors in middle school career interventions

Empirical research consistently underscores the pivotal role that counselors play in career interventions at the middle school level. This conclusion aligns with the findings of Whiston et al. (2017) in their meta-analysis, which emphasizes the significant impact of counselor support. Notably, counselor support was identified as the most critical factor influencing the effectiveness of career counseling interventions. The effectiveness of career counseling programs at the middle school stage is shaped by various factors. Van Hai et al. (2022), through qualitative research, developed a model that highlights both institutional and subjective influences. Institutional factors, such as the availability and allocation of educational resources, are crucial, while subjective factors, including the professional qualifications and dedication of administrators and teachers, also play a vital role.

To support students in making informed social and educational decisions, counselors must provide guidance on career choices that aligns with students’ interests and abilities. Effective career interventions not only offer essential information but also help students explore potential career paths, thereby fostering their problem-solving skills and decision-making abilities. These interventions serve as a foundational mechanism for nurturing middle school students’ career development, as they provide the necessary direction and support for students to make informed decisions about their futures (Isah, 2024; Sanders et al., 2017).

Future research should extend beyond examining the general effectiveness of career interventions to explore the specific strategies counselors employ in facilitating students’ career awareness and exploration. Additionally, it is crucial to investigate best practices in the professional development and training of middle school counselors, ensuring that they are adequately equipped to implement effective career development programs.

### Limitations and future directions

Intervention research primarily aims to foster career development among middle school students, with a particular emphasis on providing educational resources to special groups and reducing dropout rates. However, existing studies face several limitations. Many studies predominantly focus on special groups, such as students with learning difficulties or learning disabilities, while research on the general student population remains limited. Consequently, the applicability of these findings to typical students requires further examination.

Additionally, among the reviewed literature, only one study included a two-month follow-up assessment, making it challenging to determine the long-term sustainability of intervention effects. Furthermore, current evaluation systems rely heavily on self-reports, lacking multi-stakeholder assessments—such as those from teachers and parents—as well as objective behavioral indicators for validation. To address these gaps, future research should incorporate follow-up assessments at six and 12 months to evaluate whether students retain and generalize the skills acquired through interventions. Moreover, future studies could investigate the impact of career interventions on additional related variables, such as career adaptability. Another promising area for exploration is the integration of artificial intelligence tools into career interventions, which may enhance their effectiveness and accessibility.

Future research should incorporate objective indicators, such as academic performance, alongside evaluations from teachers and parents to improve the credibility of findings. Additionally, researchers could explore the development of a three-dimensional intervention framework that is “school-led, family-involved, and counselor-supported.” By integrating school and family interventions, tiered curricula could be designed to accommodate different student groups, thereby establishing concrete methodologies for middle school career development while also providing personalized intervention models for special groups. Furthermore, future studies should conduct comparative analyses to assess the effectiveness of different interventions across diverse student populations. Investigating career education across various cultural contexts would also be valuable in identifying universal intervention elements. Such research would contribute to a more comprehensive and globally applicable understanding of effective career interventions.

## Data Availability

The original contributions presented in the study are included in the article/supplementary material, further inquiries can be directed to the corresponding author.
